# Functional peptide therapeutics for veterinary osteoarthritis: mechanisms, delivery systems, and translational priorities

**DOI:** 10.3389/fvets.2026.1974160

**Published:** 2026-10-20

**Authors:** Soo-Cheol Choi, Min Gyeong Song, Sun Young Yi, Sung Hoon Lee, In Ah Lee

**Affiliations:** 1 Department of Chemistry, Kunsan National University, Gunsan-si, Republic of Korea; 2 Institute of Basic Science, Kunsan National University, Gunsan-si, Republic of Korea; 3 Department of Global Beauty Art, Kunsan National University, Gunsan-si, Republic of Korea; 4 Department of Pharmaceutical Engineering, Cheongju University, Cheongju-si, Republic of Korea

**Keywords:** cartilage targeting, collagen peptide, companion animals, disease-modifying osteoarthritis drug, functional peptide, intra- articular delivery, osteoarthritis, veterinary pharmacology

## Abstract

**Introduction:**

Veterinary osteoarthritis (OA) is a chronic whole-joint disorder that causes pain, gait impairment, reduced activity, and diminished quality of life in companion and performance animals. Current multimodal management includes weight control, rehabilitation, non-steroidal anti-inflammatory drugs, prostaglandin E2 EP4 receptor antagonists, anti-nerve growth factor monoclonal antibodies, intra-articular therapies, and nutraceuticals. Although these interventions improve pain and function, they generally do not restore the cartilage extracellular matrix or provide sustained disease modification. Functional peptides have emerged as promising candidates because of their potential to suppress inflammation, inhibit matrix degradation, promote chondrogenesis, target damaged cartilage, and support sustained or targeted delivery.

**Methods:**

This narrative review evaluated the pathobiological rationale, veterinary evidence, peptide classes, delivery technologies, pharmacological requirements, and translational barriers relevant to veterinary OA. Targeted literature searches and qualitative evidence appraisal focused on clinical relevance, peptide mechanisms, delivery strategies, species-specific limitations, and the distinction between nutritional collagen hydrolysates and sequence-defined therapeutic peptides.

**Results:**

Oral bioactive collagen peptides currently provide the most direct clinical evidence in naturally occurring canine and equine OA. However, the principal canine trial was limited by modest sample size and duration, predominantly symptomatic rather than structural outcomes, and manufacturer support. Thus, the available evidence supports potential adjunctive clinical benefit rather than a disease-modifying claim. Sequence-defined hyaluronan-binding, bone morphogenetic protein receptor-mimetic, collagen-binding, collagen-hybridizing, and cartilage-targeting peptides have demonstrated mechanistic or delivery advantages in cell, tissue, rodent, and selected large-animal studies, but target-species pharmacokinetic and clinical validation remains limited.

**Discussion:**

A credible development pathway should integrate target-specific activity, protease stability, immunogenicity, manufacturability, species-appropriate delivery, joint exposure, repeated-dose safety, objective gait and activity outcomes, and structural endpoints. Functional peptides are unlikely to replace multimodal care in the near term but may become valuable adjunctive platforms and contribute to future disease-modifying strategies when biological activity is supported by demonstrated joint exposure, structural protection, and rigorous comparative trials.

## 1 Introduction

Osteoarthritis is one of the most important chronic musculoskeletal disorders in veterinary practice. In a large UK primary-care population, appendicular OA was recorded in approximately 2.5% of dogs, with increasing age and body weight among the major associated factors (1). The true burden is probably higher because early disease may be underdiagnosed, clinical signs fluctuate, and owners may attribute reduced activity to normal aging. The consequences extend beyond radiographic change: persistent pain alters mobility, sleep, behavior, interaction with caregivers, and participation in normal activities.

OA is now understood as a disease of the entire synovial joint rather than simple mechanical wear of articular cartilage. Chondrocyte dysfunction, synovitis, subchondral bone remodeling, osteophyte formation, altered biomechanics, and peripheral and central sensitization interact over time (2, 3). This whole-joint perspective is especially relevant to veterinary patients, in whom developmental orthopedic disease, ligament injury, obesity, conformation, athletic loading, and aging may contribute in different proportions.

Veterinary OA treatment is therefore inherently multimodal. Analgesic and anti-inflammatory agents can improve comfort, while weight management, exercise modification, rehabilitation, and environmental adaptation reduce mechanical and functional burden. Nevertheless, a major therapeutic gap remains: available interventions are more effective at controlling symptoms than at preserving or restoring the cartilage matrix. Functional peptides are being investigated as a bridge between symptom control and disease modification because they can be designed to engage specific molecular targets, mimic extracellular-matrix signals, or direct therapeutic cargo to diseased tissue.

In this review, a disease-modifying osteoarthritis drug (DMOAD) is used in a strict translational sense to denote an intervention that modifies underlying OA pathophysiology and slows, prevents, or reverses structural joint deterioration while providing clinically meaningful benefit. Symptomatic improvement alone, including reduced pain or improved gait without evidence of altered structural progression, is therefore not considered sufficient to establish a DMOAD effect (4, 5). Because no peptide-based veterinary OA product currently satisfies this standard, sequence-defined peptides and collagen-derived products are discussed as candidates with possible disease-modifying potential rather than as established DMOADs.

This review evaluates the biological rationale and translational feasibility of functional peptide-based therapeutics for veterinary OA. The scope is source-agnostic and includes collagen hydrolysates, sequence-defined anti-inflammatory and chondroprotective peptides, receptor- or matrix-binding peptides, and peptide-enabled delivery systems; marine-derived peptides are considered only when they meet the same mechanistic and translational criteria. Emphasis is placed on evidence that can inform drug development in dogs, cats, and horses, while clearly distinguishing direct veterinary evidence from findings extrapolated from human tissues and experimental animal models.

## 2 Review approach and evidence appraisal

This narrative review was prepared using targeted PubMed/MEDLINE searches through 31 July 2026. The core veterinary OA search combined (osteoarthritis OR “degenerative joint disease”) with (dog OR canine OR cat OR feline OR horse OR equine OR veterinary). Peptide-focused searches combined (“functional peptide” OR “bioactive peptide” OR “collagen peptide” OR “collagen hydrolysate” OR “anti-inflammatory peptide” OR “chondroprotective peptide” OR “cartilage-targeting peptide” OR “collagen-binding peptide” OR “hyaluronan-binding peptide”) with (cartilage OR chondrocyte OR joint OR “intra-articular” OR hydrogel OR nanoparticle). Additional targeted searches paired the candidate terms P15-1, CK2.1, WYRGRL, and “collagen-hybridizing peptide” with osteoarthritis or cartilage. Reference lists of eligible primary studies and relevant reviews were manually checked to identify additional studies, and regulatory or product documents were consulted only for currently approved veterinary analgesic modalities.

Titles and abstracts were screened for direct relevance to veterinary OA, peptide mechanism, cartilage or synovial biology, joint targeting, formulation, pharmacokinetics, safety, or clinically relevant comparator therapy. Full texts were then reviewed when they provided original experimental or clinical data, a clearly described peptide or collagen-derived intervention, or information necessary to interpret translational development. Priority was given to peer-reviewed studies in naturally occurring veterinary OA, controlled veterinary trials, target-species mechanistic or pharmacokinetic studies, and delivery studies demonstrating joint or cartilage localization. Studies were excluded from the core evidence synthesis when they were unrelated to OA, lacked sufficient methodological detail, duplicated the same dataset, or described poorly characterized protein hydrolysates without interpretable clinical or mechanistic information. Peptide-enabled carrier studies were retained when they directly informed targeting or delivery, even when the therapeutic contribution of the peptide could not be isolated from the carrier.

Evidence was interpreted using a pragmatic four-tier translational framework rather than a formal risk-of-bias instrument. Tier 1 comprised randomized or controlled studies in animals with naturally occurring OA; Tier 2 comprised target-species mechanistic, *ex vivo*, pharmacokinetic, biodistribution, safety, or observational evidence; Tier 3 comprised induced-disease studies in laboratory or large-animal models; and Tier 4 comprised cell, biomaterial, binding, or other mechanistic studies without *in vivo* veterinary validation. The same tier definitions were applied to every peptide class. The tier reflects proximity to veterinary clinical use rather than methodological quality; within each tier, greater weight was assigned to objective functional outcomes, appropriate placebo or active comparators, predefined clinically meaningful endpoints, repeated-dose safety, and demonstration of exposure at the intended joint target.

Study selection for the final narrative synthesis was thematic rather than quantitative: eligible studies were grouped according to clinical evidence, peptide class, targeting or delivery function, pharmacological requirements, and translational barriers. No PRISMA flow diagram, pooled effect estimate, or formal meta-analysis was performed because the objective was a structured narrative appraisal of a heterogeneous field rather than a systematic review.

## 3 Veterinary osteoarthritis as a therapeutic target

### 3.1 Whole-joint pathology

Normal articular cartilage depends on a highly organized ECM rich in type II collagen and aggrecan. Type II collagen provides tensile integrity, whereas aggrecan-associated glycosaminoglycans retain water and support compressive resilience. OA develops when catabolic activity exceeds matrix synthesis and repair. Mechanical overload and inflammatory mediators activate chondrocytes and synoviocytes, promote phenotypic drift, and impair the normal anabolic program governed by SOX9, COL2A1, and ACAN.

IL-1β, TNF-α, IL-6, damaged-matrix fragments, and abnormal mechanical signals activate NF-κB and MAPK pathways. Downstream responses include increased iNOS, COX-2, NO, PGE2, MMPs, and aggrecanases. ADAMTS-5 has a central role in aggrecan loss in experimental arthritis and OA, while MMP-13 is strongly associated with type II collagen degradation and structural progression (6–8). These targets are attractive for peptide development because suppressing only pain signaling may leave the cartilage-destructive environment largely unchanged.

### 3.2 Synovitis, subchondral bone, and pain

Synovitis is present across stages of OA and can amplify cartilage catabolism through macrophage- and synoviocyte-derived cytokines. Subchondral bone undergoes regionally variable remodeling, sclerosis, microdamage, vascular invasion, and osteophyte formation. These processes alter load transfer and provide additional sources of nociceptive input. Although articular cartilage is aneural, the synovium, capsule, ligaments, periosteum, subchondral bone, and periarticular tissues are innervated.

Pain reflects a combination of inflammatory mediators, mechanical stress, NGF-dependent sensitization, and central nervous system adaptation. Consequently, a clinically meaningful peptide therapy should be evaluated at two levels: symptom modification, measured through pain and function, and structural or molecular modification, measured through joint tissues, imaging, biomarkers, or durable changes after treatment withdrawal. The pathophysiology of veterinary osteoarthritis and potential intervention points for functional peptides are summarized in Figure 1.

**Figure 1:**
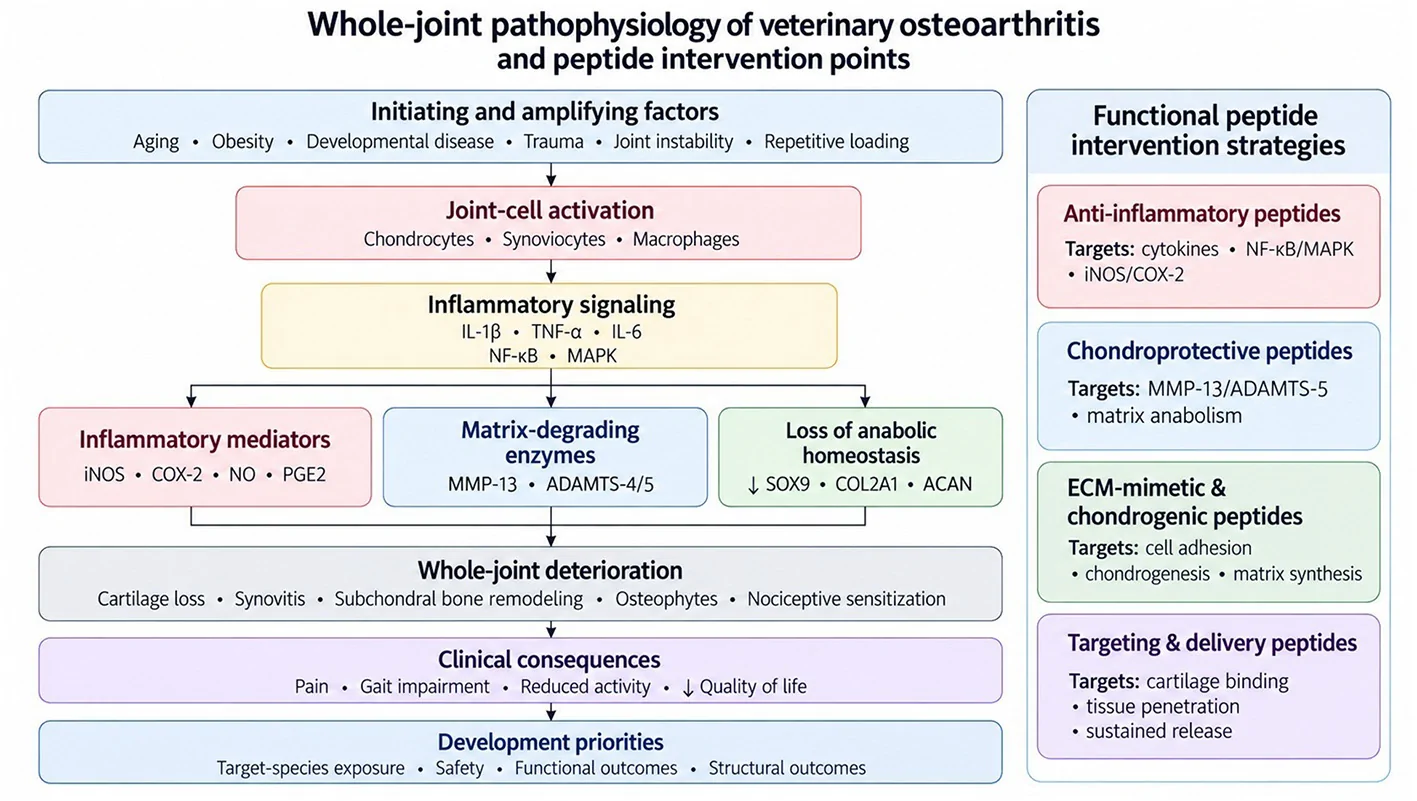
Whole-joint pathophysiology of veterinary osteoarthritis and candidate intervention points for functional peptides. Mechanical and biological triggers activate joint cells, inflammatory signaling, matrix-degrading enzymes, and pain pathways. Functional peptides may act through anti-inflammatory, chondroprotective, ECM-mimetic, chondrogenic, or tissue-targeting mechanisms.

### 3.3 Feline osteoarthritis and species-specific considerations

Feline OA warrants specific consideration because radiographic joint disease is common but clinical recognition can be difficult. Radiographic degenerative joint disease was reported in 90% of 100 cats older than 12 years, while a prospective study of 100 client-owned cats aged at least 6 years found appendicular OA in 61% and involvement of more than one joint in 48% (9, 10). Clinical signs are often expressed as gradual behavioral or lifestyle changes rather than overt lameness; owners commonly report reduced jumping, altered stair use, decreased mobility or grooming, and changes in social or exploratory behavior (10, 11). These features increase the risk of underdiagnosis and make validated owner-reported mobility measures particularly relevant in feline trials.

Peptide development for cats should therefore not simply extrapolate canine dose, exposure, palatability, or outcome measures. Feline formulations may require small dosing volumes, low-burden oral or long-acting injectable administration, and careful evaluation of species-specific metabolism, protease stability, immunogenicity, and joint exposure. Because direct feline peptide data are currently sparse, feline-specific pharmacokinetic and safety studies should precede efficacy claims, and clinical trials should combine owner-observed mobility outcomes with veterinary examination and, where feasible, objective activity measures.

## 4 Current veterinary management and the unmet need

Conventional management should remain the benchmark against which peptide therapies are judged. NSAIDs are effective for many dogs and remain central to multimodal pain management, but gastrointestinal, renal, hepatic, and hematological risks require patient selection and monitoring. Grapiprant (Galliprant^®^) acts downstream of cyclooxygenase through selective antagonism of the PGE2 EP4 receptor and has demonstrated efficacy in canine OA (12, 13). Its primary value is symptomatic control rather than restoration of joint structure.

Anti-NGF monoclonal antibodies represent a major advance in veterinary analgesia. Canine-specific anti-NGF antibodies improved pain, mobility, and function in controlled studies, and monthly bedinvetmab reduced owner-assessed OA pain in multicenter trials (14–16). In cats, the felinized anti-NGF monoclonal antibody frunevetmab improved owner- and veterinarian-assessed outcomes in a randomized placebo-controlled trial involving 275 client-owned cats with naturally occurring OA pain (17). These agents validate NGF as a clinically relevant pain target in both species, but analgesia does not establish reduced cartilage degeneration. Long-term use should therefore remain integrated with weight control, rehabilitation, environmental adaptation, and monitoring of disease progression.

Hyaluronic acid (HA) products, polysulfated glycosaminoglycans, nutraceuticals, platelet-rich plasma, and mesenchymal stromal cell-based interventions are used with variable levels of evidence and standardization. Differences in product composition, administration technique, disease stage, and outcome measures complicate comparison. A peptide-based product must offer a clear advantage: improved tolerability, structural protection, longer joint residence, lower dosing frequency, better species-specific usability, or a defined mechanism that complements existing analgesics. A comparison of established and peptide-based strategies for veterinary osteoarthritis is presented in Table 1.

**Table 1:** Comparison of established and peptide-based strategies for veterinary osteoarthritis.

Strategy	Primary action	Clinical strengths	Major limitations	DMOAD potential
NSAIDs	COX inhibition; reduced prostaglandin E2-mediated pain/inflammation	Established efficacy; oral use	GI, renal, hepatic, or hematologic risks; monitoring	Symptom-modifying
EP4 receptor antagonist (grapiprant)	Selective PGE2-EP4 blockade	Targeted oral analgesia in dogs	No proven structural benefit; GI adverse events	Symptom-modifying
Anti-NGF monoclonal antibodies (bedinvetmab; frunevetmab)	Blocks NGF-mediated nociceptor sensitization	Monthly SC dosing; effective pain control	Structural benefit unproven; pharmacovigilance needed	Pain-modifying; structural effect unproven
Hyaluronic acid (HA) and injectable joint products	Lubrication; viscoelastic and variable anti-inflammatory effects	Local delivery; generally well tolerated	Variable duration; repeated IA procedures; product heterogeneity	Supportive; inconsistent structural evidence
Nutraceuticals and collagen products	Nutritional and matrix support	Home administration; long-term use	Variable composition/dose; unclear active sequences and bioavailability	Adjunctive; structural evidence limited
Regenerative approaches	Paracrine immunomodulation; tissue repair	Potential anti-inflammatory and regenerative effects	Cost, heterogeneity, manufacturing, standardization	Potential; not established
Functional peptide therapeutics	Defined anti-inflammatory, chondroprotective, regenerative, or targeting actions	Designable; compatible with sustained or targeted delivery	Instability, immunogenicity, cost; few veterinary trials	Potential only with demonstrated structural benefit

COX, cyclooxygenase; DMOAD, disease-modifying osteoarthritis drug; EP4, prostaglandin E2 receptor 4; HA, hyaluronic acid; IA, intra-articular; NGF, nerve growth factor; NSAID, non-steroidal anti-inflammatory drug; PGE2, prostaglandin.

## 5 Functional peptide classes and therapeutic potential

The major functional peptide categories and their development priorities are summarized in Table 2.

**Table 2:** Functional peptide categories and development priorities.

Peptide category	Representative function	Preferred route/platform	Evidence tier	Key development questions
Collagen-derived peptides	Matrix support; mobility	Oral formulations	Tier 1: dogs/horses	Active sequences; exposure; dose-response; structural outcomes
Anti-inflammatory peptides	Suppress cytokine/NF-kB/MAPK signaling	Systemic, IA, or protected oral delivery	Mainly Tier 4; limited Tier 3	Protease stability; immune effects; repeated-dose safety
Chondroprotective peptides	Reduce MMP-13/ADAMTS-5; support matrix markers	IA sustained-release or targeted carriers	Tier 3-4	Cartilage preservation; durability
ECM-mimetic/chondrogenic peptides	Promote adhesion, differentiation, matrix deposition	Hydrogels, scaffolds, cell platforms	Tier 4	Fibrosis/hypertrophy; mechanical integration
Cartilage/collagen-targeting peptides	Increase joint localization/retention	Nanocarriers, HA conjugates, imaging agents	Tier 3-4; some large-animal data	Large-joint penetration; disease-stage effects; ligand density
Multifunctional peptide systems	Combine activity with targeting/self-assembly	Depots or conjugates	Tier 3-4	Manufacturing; reproducibility; regulation

ACAN, aggrecan; ADAMTS, a disintegrin and metalloproteinase with thrombospondin motifs; COX, cyclooxygenase; ECM, extracellular matrix; IA, intra-articular; iNOS, inducible nitric oxide synthase; MAPK, mitogen-activated protein kinase; MMP, matrix metalloproteinase; NF-κB, nuclear factor kappa B; NO, nitric oxide; PGE2, prostaglandin E2.

### 5.1 Collagen-derived bioactive peptides

Collagen hydrolysates are heterogeneous mixtures of low-molecular-weight peptides generated by controlled collagen hydrolysis and should be distinguished from sequence-defined therapeutic peptides, which are single molecular entities with a specified amino-acid sequence, potency assay, and pharmacokinetic profile. A collagen hydrolysate may be marketed as a nutritional supplement, but its regulatory category and evidentiary requirements depend on the intended claim; nutritional use should not be conflated with pharmacological disease modification. Collagen-derived mixtures are typically enriched in glycine, proline, and hydroxyproline. After oral administration, peptide fragments or amino-acid metabolites may be absorbed and distributed to connective tissues. Radiolabeled gelatin hydrolysate studies demonstrated accumulation of collagen-derived radioactivity in murine cartilage, supporting biological plausibility for systemic joint exposure (18). However, tissue accumulation does not identify the active sequence or prove a disease-modifying mechanism.

The most relevant veterinary evidence comes from naturally occurring disease. Specific bioactive collagen peptides improved gait variables and owner-reported quality of life in dogs with OA in a controlled clinical study (19). Related supplementation studies in horses reported improvements in mobility and lameness-related outcomes (20). These findings support oral feasibility and owner compliance, but several questions remain: whether effects are sequence specific, whether systemic concentrations are sufficient for direct chondrocyte modulation, whether benefits persist after discontinuation, and whether cartilage structure is preserved.

The 2024 canine trial provides the most detailed controlled veterinary evidence for this class to date because it combined blinded allocation, an active nutritional comparator, force-plate treadmill measurements, the Canine Brief Pain Inventory, and accelerometry in privately owned dogs. Dogs received approximately 240 ± 95 mg/kg body weight of a specific porcine type I collagen hydrolysate daily for 12 weeks, and 31 animals completed the study (19). Improvements were detected in selected kinetic variables and quality of life, whereas spontaneous activity and pain scores were not consistently improved. Interpretation requires caution: enrolled disease was predominantly mild to moderate, treatment lasted only three months, structural cartilage outcomes were not assessed, the sample was small, and the study received manufacturer support with disclosed commercial relationships. The findings therefore establish a clinically relevant adjunctive signal but do not demonstrate delayed structural progression or justify class-wide extrapolation to all collagen hydrolysates.

For product development, collagen-derived peptides should be characterized by source species, hydrolysis process, molecular-weight distribution, marker sequences, contaminant profile, batch-to-batch bioactivity, dose, and palatability. A therapeutic claim requires more than symptomatic improvement; it should link the product to validated molecular, imaging, or structural endpoints. Key veterinary clinical studies of collagen-derived peptides are summarized in Table 3.

**Table 3:** Key veterinary clinical studies of collagen-derived peptide interventions in naturally occurring osteoarthritis.

Study/species	Sample size and groups	Intervention/comparator/duration	Outcomes and findings	Key limitations/tier
Bioactive collagen peptides (BCP); client-owned dogs with spontaneous OA (19)	*n* = 31: BCP 11; omega-3 + vitamin E 11; placebo 9	~240 ± 95 mg/kg PO daily; active comparator + placebo; 12 wk	Force plate, CBPI, activity: selected gait and QoL improved; pain/activity inconsistent	Small, short study; mild-moderate OA; no structural endpoint; industry support. Tier 1
PETAGILE BCP; privately owned horses with mild-moderate OA (20)	*n* = 38; 25 or 50 g/day; placebo available at one center	Oral BCP 25 or 50 g/day; placebo in one center; 12 wk	Orthopedic exams + owner scores: several lameness, flexion-pain, and mobility measures improved	Pilot two-center design; unequal comparator structure; small groups; subjective outcomes; no structural endpoint. Tier 1

BCP, bioactive collagen peptides; CBPI, Canine Brief Pain Inventory; n3FA, omega-3 fatty acids plus vitamin E; OA, osteoarthritis; QoL, quality of life.

### 5.2 Anti-inflammatory and chondroprotective peptides

Sequence-defined anti-inflammatory peptides are intended to interrupt cytokine signaling, suppress NF-κB or MAPK activation, reduce iNOS and COX-2 expression, or modulate macrophage phenotype. Chondroprotective peptides additionally aim to inhibit MMP-13 or ADAMTS-5 while maintaining SOX9, COL2A1, and ACAN. The distinction matters because reduction of inflammatory markers alone does not establish protection of cartilage.

The hyaluronan-binding peptide P15-1 illustrates a multifunctional approach. In IL-1β-stimulated human articular chondrocytes, P15-1 combined with high-molecular-weight HA reduced inflammatory and catabolic responses and supported the pericellular matrix, partly through p38 MAPK modulation (21). This type of peptide may be particularly suited to IA formulations in which HA contributes lubrication, retention, and a biological cofactor. Nevertheless, veterinary translation requires testing in canine, feline, or equine cells and in naturally occurring disease.

A complementary regenerative example is CK2.1, a bone morphogenetic protein receptor type IA-mimetic peptide designed to activate downstream signaling by disrupting receptor association with casein kinase 2. In a murine destabilization-of-the-medial-meniscus model, CK2.1 was linked to hyaluronic-acid hydrogel particles for approximately seven-day release and administered intra-articularly at 6 μM twice at two-week intervals. The system increased type II and IX collagen and reduced hypertrophic type X collagen relative to controls (22). This study demonstrates how peptide mechanism and depot formulation can be co-developed, but it did not establish analgesia, target-species pharmacokinetics, or efficacy in naturally occurring veterinary disease.

Candidate selection should prioritize concentration-dependent efficacy within a clearly non-cytotoxic range. A useful screening panel includes cytokine release, NO and PGE2 production, NF-κB/MAPK activation, MMP-13 and ADAMTS-5, matrix synthesis markers, apoptosis, oxidative stress, and preservation of proteoglycan in cartilage explants. Peptides that reduce inflammation but impair cell viability or anabolic function should be excluded early.

### 5.3 Extracellular-matrix-mimetic and chondrogenic peptides

ECM-mimetic peptides reproduce cell-binding or matrix-binding motifs and are usually developed as components of scaffolds, hydrogels, or cell therapies rather than as freely soluble drugs. RGD-containing motifs can enhance integrin-mediated adhesion, whereas collagen-mimetic, HA-binding, or chondroitin sulfate-binding sequences can create a microenvironment favorable to chondrogenesis. Collagen-mimetic peptide-modifiable hydrogels have supported chondrogenic differentiation and cartilage-like matrix formation in experimental systems (23).

The principal translational risk is loss of biological specificity. Strong adhesion is not automatically regenerative and may favor fibrocartilage, fibrosis, hypertrophy, or inappropriate synovial responses. Studies should therefore quantify not only SOX9, type II collagen, and aggrecan, but also type I collagen, type X collagen, alkaline phosphatase, and fibrotic markers. Mechanical properties and integration with native cartilage are as important as histological appearance.

### 5.4 Cartilage-targeting and collagen-binding peptides

Joint targeting addresses a central delivery problem: small peptides can be cleared rapidly from synovial fluid, while dense, negatively charged cartilage limits penetration. Active targeting peptides can bind type II collagen, damaged collagen, aggrecan-associated structures, or receptors on chondrocytes and synoviocytes. Their value may be intrinsic or may arise from conjugation to nanoparticles, liposomes, polymers, HA, imaging agents, or therapeutic proteins.

Type II collagen-binding motifs have increased the retention and biodistribution of nanoparticles in cartilage, although targeting performance changes with disease severity and matrix composition (24). A WYRGRL-modified nanoplatform improved cartilage-directed delivery in experimental OA (25). Collagen-binding peptides identified through screening have also been used to enhance HA retention, lubrication, imaging, and regenerative cell homing in osteoarthritic cartilage (26). Collagen-hybridizing peptides, which recognize unfolded collagen chains, extended joint retention in experimental IA delivery studies and may preferentially localize to sites of active matrix damage (27).

Targeting performance is not a fixed property of a peptide sequence. Cartilage charge density, collagen exposure, matrix porosity, synovial inflammation, and lesion depth change with disease stage and can alter binding and penetration. WYRGRL, a type II collagen-binding hexapeptide, enhanced cartilage localization of a multifunctional nanocarrier and delayed degeneration in a rat anterior cruciate ligament transection model (25). By contrast, phage-display-derived collagen-binding peptides containing a WXPXW consensus motif bound collagen type XII and enhanced retention of hyaluronic-acid constructs in *ex vivo* human cartilage, rats, and minipigs (26). Collagen-hybridizing peptides recognize unfolded collagen chains and prolonged intra-articular retention of a fluorescent payload in rat knees (27). These systems provide strong proof of concept, but the therapeutic contribution of the targeting peptide must be distinguished from that of the attached drug, cell, carrier, or physical stimulus.

These findings are highly relevant to veterinary development because large-animal joints create different diffusion distances, synovial volumes, loading conditions, and clearance kinetics than rodent joints. A targeting ligand that performs well in mice should therefore be re-evaluated in canine or equine cartilage, synovial fluid, and joint explants before expensive clinical development. Representative sequence-defined peptide and peptide-enabled systems are summarized in Table 4.

**Table 4:** Representative sequence-defined peptide and peptide-enabled systems relevant to veterinary osteoarthritis translation.

Candidate/system	Model/species	Exposure/platform	Main findings	Evidence tier/key limitation
P15-1 + high-MW HA (21)	IL-1beta-stimulated human chondrocytes	P15-1 with high-MW HA	Reduced inflammatory/catabolic responses; supported pericellular matrix	Tier 4; no veterinary PK, safety, or *in vivo* efficacy
CK2.1 BMP receptor-mimetic peptide (22)	Murine DMM OA	6 μM IA in HA hydrogel; 2 injections	Increased collagen II/IX; reduced collagen X	Tier 3; no target-species PK, analgesic, or spontaneous-disease data
WYRGRL cartilage-targeting nanocarrier (25)	Rat ACLT OA	IA carrier with bilirubin + rapamycin	Improved cartilage localization; delayed degeneration	Tier 3; peptide effect confounded by drugs/carrier; complex manufacture
WXPXW collagen-binding peptide (26)	Human OA cartilage; rat/minipig OA	Peptide-modified HA constructs	Improved retention, lubrication, cell homing, regeneration	Tier 3-4; no spontaneous veterinary OA or stand-alone efficacy
Collagen-hybridizing peptide (27)	Rat knee	IA fluorophore conjugate	Bound damaged collagen; prolonged payload retention	Tier 3; therapeutic efficacy and repeated-dose veterinary safety unknown

HA, hyaluronic acid; IA, intra-articular; OA, osteoarthritis; PK, pharmacokinetics, ACLT, anterior cruciate ligament transection; BMP, bone morphogenetic protein; DMM.

## 6 Translational development strategy

### 6.1 Candidate discovery and stage-gated selection

Discovery can begin with natural-protein hydrolysates, rationally designed sequences, known receptor-binding motifs, phage display, or computational screening. The first go/no-go decision should integrate potency with developability. A candidate with modest potency but high stability, low immunogenicity, and economical synthesis may be more viable than a highly active sequence that is rapidly degraded or difficult to manufacture. These developability constraints are well recognized across peptide-therapeutic programs (28).

Minimum early-stage criteria should include verified identity and purity, reproducible biological activity, lack of cytotoxicity, serum and synovial-fluid stability, preliminary species cross-reactivity, and compatibility with the intended formulation. Where possible, activity should be confirmed in primary cells or explants from the target species rather than relying exclusively on immortalized human or rodent cells.

### 6.2 Route of administration

Oral administration offers the greatest convenience for long-term management and is commercially attractive for dogs and horses. It is most plausible for collagen-derived or food-derived peptides that tolerate partial gastrointestinal degradation or whose metabolites retain activity. Development should measure digestive stability, intestinal permeability, systemic exposure, metabolites, joint distribution, and palatability. Enteric protection, encapsulation, permeation enhancers, or sequence modification may improve exposure but can complicate classification and cost.

Subcutaneous or intramuscular dosing bypasses gastrointestinal degradation and can support long-acting formulations. Systemic injection is appropriate when multiple joints are affected, but renal clearance, serum proteolysis, injection-site reactions, and anti-peptide antibodies must be monitored. Half-life extension through lipidation, albumin binding, PEGylation, cyclization, terminal modification, D-amino-acid substitution, or depot formulation may improve dosing intervals, although each modification can alter potency and distribution.

IA administration provides high local exposure and is attractive for peptides that act on cartilage, synovium, or the pericellular matrix. The disadvantages are procedural burden, infection risk, rapid synovial clearance, and potential need for sedation. IA products should therefore be designed for sustained release or tissue binding rather than as simple aqueous solutions whenever the peptide half-life is short.

### 6.3 Formulation and delivery systems

HA-, gelatin-, collagen-, chitosan-, alginate-, or polyethylene glycol-based hydrogels can provide local retention and controlled release. Thermosensitive or *in situ* cross-linking systems may be injected through a needle and gel within the joint. Nanoparticles, liposomes, microspheres, and biodegradable polymer depots can protect peptides from proteases and tune release kinetics. The optimal system depends on whether rapid suppression of inflammation, prolonged matrix protection, or regenerative signaling is required.

Physicochemical properties should be linked to biological performance. Particle size, charge, ligand density, encapsulation efficiency, gelation time, mechanical properties, burst release, degradation, and injectability influence joint distribution and safety. Positively charged carriers may penetrate negatively charged cartilage but can also increase cytotoxicity. Excessively persistent materials may produce synovitis or interfere with joint mechanics. Formulation screening should therefore include synovial-cell compatibility and joint explant testing, not only release curves.

### 6.4 Preclinical efficacy, pharmacokinetics, and clinical outcomes

Evaluation should progress from cells to tissues, induced OA models, and naturally occurring disease. *In vitro* studies should use inflammatory stimulation of chondrocytes, synoviocytes, or macrophages and report both catabolic and anabolic markers. *Ex vivo* cartilage or osteochondral explants provide a more realistic diffusion barrier and permit measurement of glycosaminoglycan release, collagen degradation, histology, and matrix penetration.

Rodent MIA models are efficient for pain and inflammatory readouts, whereas surgical instability models better represent progressive structural disease. OARSI-compatible histology improves comparability across studies (29). However, successful rodent studies should be followed by target-species studies because joint size, gait, cartilage thickness, and metabolism affect exposure and response. For IA products, synovial-fluid concentration, cartilage penetration, and residence time should be measured directly.

Pharmacokinetic characterization is a major unresolved requirement for veterinary peptide development. After IA administration, small biologics may be removed rapidly across the synovial membrane, while diffusion into avascular cartilage is constrained by molecular size, charge, binding affinity, matrix density, and disease-associated changes in permeability (30). Intact-peptide stability should therefore be measured in species-specific synovial fluid as well as plasma, because local proteases, protein binding, and inflammatory composition can alter degradation independently of systemic half-life. Interspecies extrapolation is particularly uncertain: dogs, cats, horses, and rodents differ in joint volume, cartilage thickness, synovial surface area, loading, lymphatic clearance, and feasible IA dose volume. Measurement of intra-articular exposure is also technically challenging because repeated arthrocentesis can perturb joint homeostasis, synovial-fluid concentration may not reflect intratissue cartilage concentration, and labeled constructs may report carrier or metabolite rather than intact active peptide. Where feasible, LC-MS/MS quantification of intact peptide and metabolites should be combined with cartilage or synovial tissue measurements, biodistribution imaging, and exposure-response analysis to establish whether the target tissue remains above the active concentration for the intended dosing interval.

Clinical relevance requires objective function. Force-plate analysis, pressure-sensitive walkways, wearable activity monitors, range of motion, and validated owner-reported instruments should be integrated. The CBPI can detect treatment response in canine OA and is useful when combined with objective measures (31). Pain improvement alone should not be interpreted as disease modification; imaging, biomarkers, or structural endpoints are necessary for a DMOAD claim.

When dogs are used as the target species or as a translational large-animal model, histopathological evaluation should follow species-specific OARSI recommendations rather than relying solely on murine scoring conventions (32). Structural endpoints should be linked to synovial-fluid or tissue exposure, because a negative efficacy result may reflect inadequate joint concentration rather than failure of the molecular mechanism.

### 6.5 Safety, quality, and regulatory positioning

Peptides are often perceived as intrinsically safe, but repeated exposure can produce immune responses, local irritation, off-target receptor activity, complement activation, or organ toxicity. Safety programs should include clinical pathology, injection-site evaluation, anti-drug antibodies, cytokine responses, major-organ histology, and species-appropriate reproductive or residue considerations when relevant. IA products require special attention to sterility, endotoxin, particles, pH, osmolality, and synovial tolerance.

CMC development should define sequence, molecular mass, purity, related substances, oxidation, deamidation, truncation products, residual solvents, counterions, water content, potency, and stability. Analytical methods may include HPLC, LC-MS/MS, MALDI-TOF MS, amino-acid analysis, and a mechanism-linked bioassay. Formulated products additionally require specifications for particle size, zeta potential, encapsulation, release, gelation, viscosity, container compatibility, and microbial quality.

Regulatory positioning should be decided early. A product marketed solely for nutritional joint support may follow a different pathway from a product claiming analgesia, suppression of inflammation, cartilage protection, or delayed progression. The intended claim determines the required evidence, manufacturing controls, and clinical design. Early consultation with the relevant veterinary regulatory authority can prevent late-stage redevelopment.

Regulatory strategy should therefore begin with a target product profile rather than after efficacy has been demonstrated. A nutritional supplement, a locally administered veterinary medicinal product, and a peptide-functionalized combination product require different evidence packages. Sequence-defined candidates should have release specifications for identity, purity, related substances, potency, sterility where applicable, and stability. Hydrolysates should additionally define source traceability, molecular-weight distribution, marker-peptide ranges, contaminant control, and batch-level bioactivity. Early dialogue with the relevant authority is particularly important for products combining a peptide with a device-like hydrogel, nanoparticle, cell product, or imaging function. The proposed stage-gated translational development pathway for functional peptide therapeutics is illustrated in Figure 2. The recommended development framework and outcome measures are summarized in Table 5.

**Figure 2:**
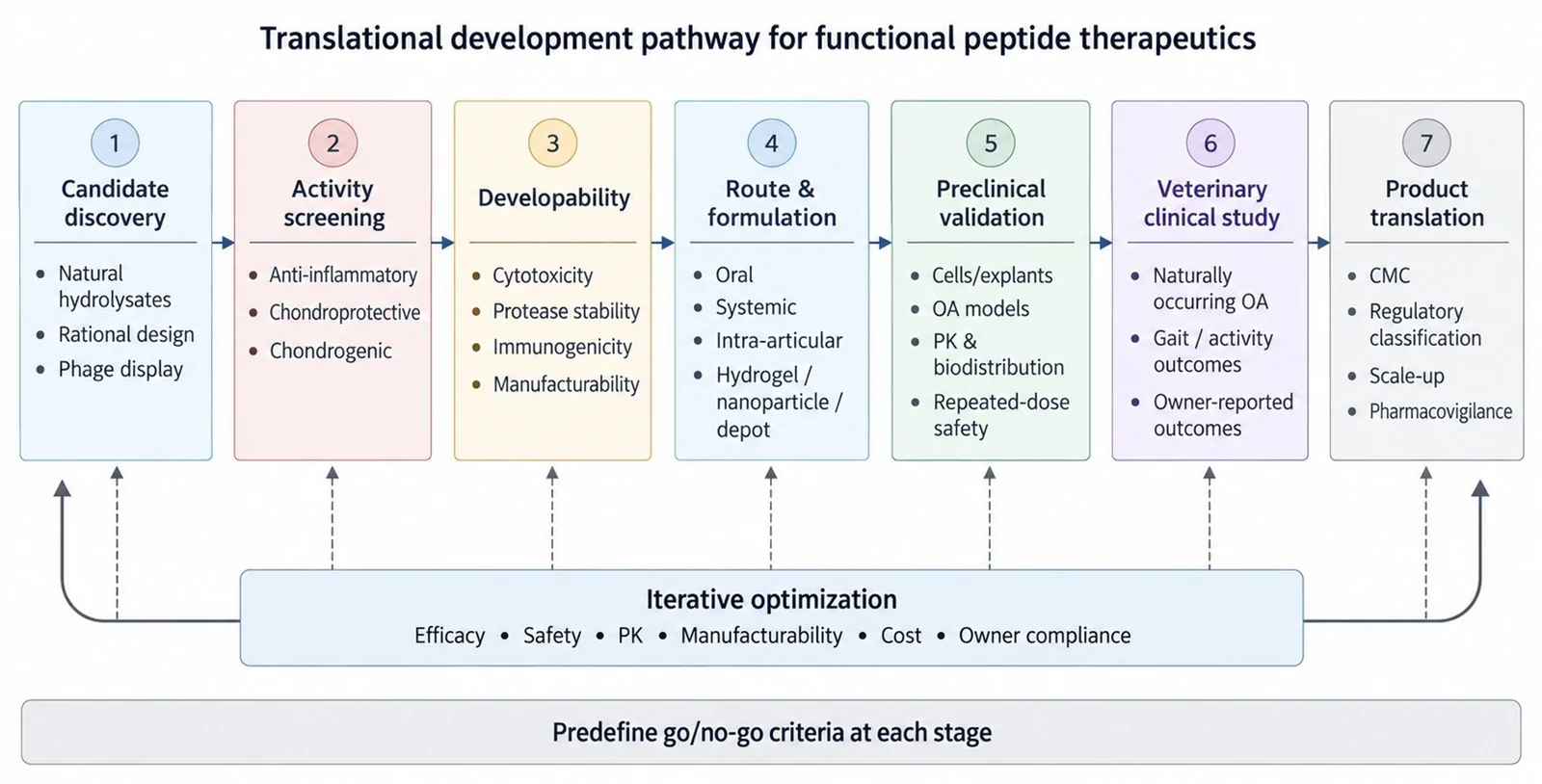
Stage-gated translational pathway for functional peptide therapeutics. Candidate discovery should proceed in parallel with developability, route selection, formulation, pharmacokinetics, safety, veterinary clinical validation, and CMC planning. Iterative go/no-go decisions reduce the risk of advancing biologically interesting but commercially or clinically unsuitable candidates.

**Table 5:** Recommended development framework and outcome measures.

Development stage	Recommended model/assay	Core outcomes	Go/no-go criterion
Identity/activity	Analytical + target/cell assay	Identity, purity, potency, impurities	Reproducible activity across batches
Cell screening	Target-species joint cells/macrophages	Viability; inflammatory, catabolic, anabolic markers	Efficacy at non-cytotoxic concentrations
Tissue validation	Cartilage/synovial/osteochondral explants	Penetration; matrix loss; histology	Activity retained in intact tissue
PK/delivery	Plasma, synovial fluid, cartilage	Half-life; exposure; biodistribution; residence	Joint exposure exceeds active level for intended interval
Induced OA model	MIA or surgical instability	Pain, gait, histology, imaging, biomarkers	Concordant functional/structural benefit
Naturally occurring OA	Client-owned dogs, cats, or horses	Gait/activity; owner outcomes; rescue use; safety	Clinically meaningful benefit vs comparator
CMC/product stability	Pilot and scale-up batches	Potency; degradation; sterility; shelf life	Manufacturable, stable dosage form

ACAN, aggrecan; ADAMTS, a disintegrin and metalloproteinase with thrombospondin motifs; CMC, chemistry, manufacturing, and controls; IA, intra-articular; MIA, monosodium iodoacetate; MMP, matrix metalloproteinase; OA, osteoarthritis; PK, pharmacokinetics.

## 7 Discussion

The available literature supports functional peptides as a technically versatile but clinically immature therapeutic class for veterinary osteoarthritis. The field has progressed from general collagen supplementation toward sequence-defined ligands, receptor-mimetic peptides, matrix-binding motifs, and multifunctional peptide–carrier systems. Nevertheless, the evidence remains uneven: direct veterinary clinical data are concentrated in collagen hydrolysates, whereas the most sophisticated sequence-defined systems have largely been evaluated in human cells, rodents, or experimentally induced disease. This mismatch creates several controversies that should be resolved before disease-modifying claims are advanced.

### 7.1 Evidence hierarchy and central controversies

First, “functional peptide” is used for materially different products. Collagen hydrolysates are heterogeneous mixtures characterized by source, hydrolysis process, molecular-weight distribution, and marker peptides; sequence-defined therapeutics are single molecular entities with an assigned sequence, potency assay, and pharmacokinetic profile. Evidence for one category should not be generalized to the other. Second, symptomatic improvement is not equivalent to disease modification. Better gait or owner-reported quality of life may reflect analgesia, altered activity, or placebo effects unless accompanied by prespecified imaging, biomarker, or histological evidence of slower structural progression. Third, high local retention is not necessarily beneficial if a carrier limits cartilage penetration, produces a burst concentration above the safe range, or persists long enough to provoke synovitis. Finally, increasingly complex peptide–nanoparticle–hydrogel combinations may improve efficacy but simultaneously increase manufacturing variability, analytical burden, cost, and regulatory uncertainty.

A practical interpretation is that oral collagen-derived products currently have the shortest route to adjunctive veterinary use, while sequence-defined anti-inflammatory, regenerative, and targeting peptides have the stronger long-term potential to become regulated therapeutics. Advancement of either group requires direct comparison with established care, transparent reporting of industry involvement, replication by independent groups, and outcome measures that match the intended claim. For a pain-relief claim, clinically meaningful improvement in validated pain and function measures is central. For a disease-modifying claim, structural preservation, durable benefit after treatment withdrawal, and exposure–response relationships should be demonstrated in addition to symptomatic benefit.

### 7.2 Translational priorities: species, exposure, and multimodal integration

Most sequence-defined peptide studies use human chondrocytes, murine OA models, or engineered biomaterials. Dogs, cats, and horses differ in cartilage thickness, joint volume, metabolism, immunogenicity, disease etiology, feasible dosing, and the clinical outcomes that can be measured reliably. Direct cross-species extrapolation is therefore uncertain, and early access to target-species cells, synovial fluid, explants, and naturally occurring cases should be built into development programs. This is particularly relevant in cats, where OA is common but clinical signs are often behavior-based and direct peptide evidence is especially limited.

Mechanistic development should identify the receptor, binding partner, intracellular target, or minimum active motif whenever possible, because structure-activity relationships support potency optimization, safety assessment, patentability, and quality control. For hydrolysates, marker peptides and orthogonal bioassays are needed to connect batch composition with efficacy. Exposure is equally critical: plasma concentration alone may be insufficient when the relevant compartment is cartilage, synovium, or synovial fluid. Large-animal studies should quantify intact peptide, metabolites, target binding, cartilage penetration, and residence time, while targeted-carrier studies should determine how ligand density and disease stage affect surface binding, matrix penetration, and synovial clearance.

Clinical trials should enroll well-characterized naturally occurring OA cases and stratify by affected joint, disease stage, body condition, concurrent medication, and activity. Randomization, masking, placebo or active comparators, rescue-medication rules, predefined treatment-success criteria, and clinically meaningful effect sizes are required. Objective gait or activity measures should be paired with validated owner-reported outcomes, while structural claims require imaging, biomarkers, histology where feasible, or follow-up sufficient to distinguish transient analgesia from altered progression.

The most realistic near-term application is likely to be multimodal add-on therapy rather than replacement of established care. A peptide could be evaluated alongside standardized weight management and rehabilitation, combined with symptom-modifying agents such as NSAIDs, EP4 antagonists, bedinvetmab or frunevetmab, incorporated into regenerative biomaterial or cell-based strategies, or linked to advanced HA-, hydrogel-, nanoparticle-, or collagen-targeted delivery systems. Potential advantages include reduced rescue-analgesic use, lower doses of established drugs, longer intervals between IA procedures, improved local retention, or simultaneous targeting of matrix degradation while an established therapy controls pain. However, additive efficacy and safety should be demonstrated prospectively rather than inferred from mechanistic complementarity.

Add-on, factorial, or adaptive trial designs may help determine whether peptide therapy increases responder rates or benefits phenotypic subgroups defined by joint, disease stage, inflammatory features, body condition, or baseline activity. Such designs should retain a standardized background of permitted care and prespecified rescue-medication rules so that any incremental peptide effect can be interpreted clinically.

### 7.3 Limitations of the current evidence base

This Review is narrative rather than systematic and does not provide a quantitative pooled effect estimate. The peptide literature is heterogeneous in terminology, product composition, disease models, routes, carriers, and endpoints. Publication bias is likely because many studies report positive proof-of-concept findings in small experimental cohorts, and independent replication is uncommon. Direct evidence in cats is especially limited. These limitations prevent ranking individual candidates by efficacy and require cautious extrapolation from human and rodent research to veterinary patients.

## 8 Conclusion

Functional peptides offer a versatile route to expand veterinary OA treatment beyond analgesia. Their potential derives from the ability to combine anti-inflammatory activity, inhibition of cartilage-degrading enzymes, support of matrix anabolism, regenerative signaling, and tissue targeting. Oral bioactive collagen peptides already have clinically relevant evidence in naturally occurring canine and equine OA, whereas sequence-defined anti-inflammatory, ECM-mimetic, collagen-binding, and cartilage-targeting peptides remain primarily preclinical.

Successful translation will depend less on demonstrating another reduction in an inflammatory marker and more on integrating mechanism, exposure, formulation, safety, manufacturing, and clinically meaningful function. The strongest candidates will show reproducible activity in target-species systems, sufficient joint exposure, compatibility with chronic administration, objective improvement in naturally occurring OA, and evidence of structural protection when disease-modifying potential is proposed. Functional peptides should therefore be developed as components of a stage-gated veterinary therapeutic strategy rather than as generic joint supplements.

## Data Availability

The original contributions presented in the study are included in the article/supplementary material, further inquiries can be directed to the corresponding author.

## References

[1] Anderson KL O'Neill DG Brodbelt DC Church DB Meeson RL Sargan D . Prevalence, duration and risk factors for appendicular osteoarthritis in a UK dog population under primary veterinary care. Sci Rep. (2018) 8:5641. doi: 10.1038/s41598-018-23940-z 29618832 PMC5884849

[2] Loeser RF Goldring SR Scanzello CR Goldring MB. Osteoarthritis: a disease of the joint as an organ. Arthritis Rheum. (2012) 64:1697–707. doi: 10.1002/art.34453 22392533 PMC3366018

[3] Johnston SA. Osteoarthritis: joint anatomy, physiology, and pathobiology. Vet Clin North Am Small Anim Pract. (1997) 27:699–723. doi: 10.1016/S0195-5616(97)50076-3 9243777

[4] Cooper C Adachi JD Bardin T Berenbaum F Flamion B Jonsson H . How to define responders in osteoarthritis. Curr Med Res Opin. (2013) 29:719–29. doi: 10.1185/03007995.2013.792793 23557069 PMC3690437

[5] Oo WM Hunter DJ. Disease modification in osteoarthritis: are we there yet? Clin Exp Rheumatol. (2019) 37:S135–40. 31621568

[6] Glasson SS Askew R Sheppard B Carito B Blanchet T Ma HL . Deletion of active ADAMTS5 prevents cartilage degradation in a murine model of osteoarthritis. Nature. (2005) 434:644–8. doi: 10.1038/nature03369 15800624

[7] Stanton H Rogerson FM East CJ Golub SB Lawlor KE Meeker CT . ADAMTS5 is the major aggrecanase in mouse cartilage *in vivo* and *in vitro*. Nature. (2005) 434:648–52. doi: 10.1038/nature03417 15800625

[8] Wang M Sampson ER Jin H Li J Ke QH Im HJ . MMP13 is a critical target gene during the progression of osteoarthritis. Arthritis Res Ther. (2013) 15:R5. doi: 10.1186/ar4133 23298463 PMC3672752

[9] Hardie EM Roe SC Martin FR. Radiographic evidence of degenerative joint disease in geriatric cats: 100 cases (1994–1997). J Am Vet Med Assoc. (2002) 220:628–32. doi: 10.2460/javma.2002.220.628 12418522

[10] Slingerland LI Hazewinkel HAW Meij BP Picavet P Voorhout G. Cross-sectional study of the prevalence and clinical features of osteoarthritis in 100 cats. Vet J. (2011) 187:304–9. doi: 10.1016/j.tvjl.2009.12.014 20083417

[11] Klinck MP Frank D Guillot M Troncy E. Owner-perceived signs and veterinary diagnosis in 50 cases of feline osteoarthritis. Can Vet J. (2012) 53:1181–6. 23633711 PMC3474573

[12] Rausch-Derra LC Huebner M Wofford J Rhodes L A. prospective, randomized, masked, placebo-controlled multisite clinical study of grapiprant, an EP4 prostaglandin receptor antagonist, in dogs with osteoarthritis. J Vet Intern Med. (2016) 30:756–63. doi: 10.1111/jvim.13948 27075237 PMC4913586

[13] Shaw KK Rausch-Derra LC Rhodes L. Grapiprant: an EP4 prostaglandin receptor antagonist and novel therapy for pain and inflammation. Vet Med Sci. (2016) 2:3–9. doi: 10.1002/vms3.13 29067176 PMC5645826

[14] Lascelles BDX Knazovicky D Case B Freire M Innes JF Drew AC . A canine-specific anti-nerve growth factor antibody alleviates pain and improves mobility and function in dogs with degenerative joint disease-associated pain. BMC Vet Res. (2015) 11:101. doi: 10.1186/s12917-015-0413-x 25926287 PMC4419463

[15] Corral MJ Moyaert H Fernandes T Escalada M Tena JKS Walters RR . A prospective, randomized, blinded, placebo-controlled multisite clinical study of bedinvetmab, a canine monoclonal antibody targeting nerve growth factor, in dogs with osteoarthritis. Vet Anaesth Analg. (2021) 48:943–55. doi: 10.1016/j.vaa.2021.08.001 34565678

[16] Michels GM Honsberger NA Walters RR Tena JKS Cleaver DM. A prospective, randomized, double-blind, placebo-controlled multisite, parallel-group field study in dogs with osteoarthritis evaluating bedinvetmab, a canine anti-nerve growth factor monoclonal antibody. Vet Anaesth Analg. (2023) 50:446–58. doi: 10.1016/j.vaa.2023.06.003 37541934

[17] Gruen ME Myers JAE Tena JKS Becskei C Cleaver DM Lascelles BDX. Frunevetmab, a felinized anti-nerve growth factor monoclonal antibody, for the treatment of pain from osteoarthritis in cats. J Vet Intern Med. (2021) 35:2752–62. doi: 10.1111/jvim.16291 34724255 PMC8692178

[18] Oesser S Adam M Babel W Seifert J. Oral administration of 14C-labeled gelatin hydrolysate leads to an accumulation of radioactivity in cartilage of mice. J Nutr. (1999) 129:1891–5. doi: 10.1093/jn/129.10.1891 10498764

[19] Dobenecker B Böswald LF Reese S Steigmeier-Raith S Trillig L Oesser S . The oral intake of specific bioactive collagen peptides improves gait and quality of life in canine osteoarthritis patients: a translational large-animal model for a nutritional therapy option. PLoS One. (2024) 19:e0308378. doi: 10.1371/journal.pone.0308378 39298537 PMC11412516

[20] Dobenecker B Reese S Jahn W Schunck M Hugenberg J Louton H . Specific bioactive collagen peptides as a supplement for horses with osteoarthritis: a two-centred study. J Anim Physiol Anim Nutr. (2018) 102:16–23. doi: 10.1111/jpn.12863 29623685

[21] Shortt C Zhang C Snyder A Wu Y McNulty AL Guilak F . A hyaluronan-binding peptide (P15-1) reduces inflammatory and catabolic events in interleukin-1β-treated human articular chondrocytes. Sci Rep. (2020) 10:1441. doi: 10.1038/s41598-020-57586-7 31996703 PMC6989647

[22] Akkiraju H Srinivasan PP Xu X Jia X Kirn Safran CB. Nohe A. CK21, a bone morphogenetic protein receptor type IA mimetic peptide, repairs cartilage in mice with destabilized medial meniscus. Stem Cell Res Ther. (2017) 8:82. doi: 10.1186/s13287-017-0537-y 28420447 PMC5395786

[23] Parmar PA Chow LW St-Pierre JP Horejs CM Peng YY Werkmeister JA . Collagen-mimetic peptide-modifiable hydrogels for articular cartilage regeneration. Biomaterials. (2015) 54:213–25. doi: 10.1016/j.biomaterials.2015.02.079 25907054 PMC4416732

[24] Brown SB Wang L Jungels RR Sharma B. Effects of cartilage-targeting moieties on nanoparticle biodistribution in healthy and osteoarthritic joints. Acta Biomater. (2020) 101:469–83. doi: 10.1016/j.actbio.2019.10.003 31586725 PMC7025912

[25] Xue S Zhou X Sang W Wang C Lu H Xu Y . Cartilage-targeting peptide-modified dual-drug delivery nanoplatform with near-infrared laser response for osteoarthritis therapy. Bioact Mater. (2021) 6:2372–89. doi: 10.1016/j.bioactmat.2021.01.017 33553822 PMC7844135

[26] Lin CY Wang YL Chen YJ Ho CT Chi YH Chan LY . Collagen-binding peptides for the enhanced imaging, lubrication and regeneration of osteoarthritic articular cartilage. Nat Biomed Eng. (2022) 6:1105–17. doi: 10.1038/s41551-022-00948-5 36229661

[27] Luke EN Ratnatilaka Na Bhuket P Yu SM Weiss JA. Targeting damaged collagen for intra-articular delivery of therapeutics using collagen hybridizing peptides. J Orthop Res. (2023) 41:2424–32. doi: 10.1002/jor.25577 37087677 PMC10590823

[28] Fosgerau K Hoffmann T. Peptide therapeutics: current status and future directions. Drug Discov Today. (2015) 20:122–8. doi: 10.1016/j.drudis.2014.10.003 25450771

[29] Glasson SS Chambers MG Van Den Berg WB Little CB. The OARSI histopathology initiative: recommendations for histological assessments of osteoarthritis in the mouse. Osteoarthritis Cartilage. (2010) 18:S17–23. doi: 10.1016/j.joca.2010.05.025 20864019

[30] Siefen T Bjerregaard S Borglin C Lamprecht A. Assessment of joint pharmacokinetics and consequences for the intraarticular delivery of biologics. J Control Release. (2022) 348:745–59. doi: 10.1016/j.jconrel.2022.06.015 35714731

[31] Brown DC Boston RC Coyne JC Farrar JT. Ability of the canine brief pain inventory to detect response to treatment in dogs with osteoarthritis. J Am Vet Med Assoc. (2008) 233:1278–83. doi: 10.2460/javma.233.8.1278 19180716 PMC2896492

[32] Cook JL Kuroki K Visco D Pelletier JP Schulz L Lafeber FPJG. The OARSI histopathology initiative: recommendations for histological assessments of osteoarthritis in the dog. Osteoarthritis Cartilage. (2010) 18:S66–79. doi: 10.1016/j.joca.2010.04.017 20864024

